# Volatile Profile of Two-Phase Olive Pomace (Alperujo) by HS-SPME-GC–MS as a Key to Defining Volatile Markers of Sensory Defects Caused by Biological Phenomena in Virgin Olive Oil

**DOI:** 10.1021/acs.jafc.1c01157

**Published:** 2021-04-27

**Authors:** Lorenzo Cecchi, Marzia Migliorini, Elisa Giambanelli, Anna Cane, Nadia Mulinacci, Bruno Zanoni

**Affiliations:** † Department of NEUROFARBA, University of Florence, Via Ugo Schiff 6, 50019 Sesto F.no, Florence, Italy; ‡ Carapelli Firenze S.p.A., Via Leonardo da Vinci 31, Tavarnelle Val di Pesa, 50028 Firenze, Italy; § Department of Agricultural, Food and Forestry Systems Management (DAGRI), University of Florence, Piazzale Delle Cascine 16, 50144 Florence, Italy

**Keywords:** virgin olive oil classification, panel test, olive oil volatile organic compounds, olive oil by-products, HS-SPME-GC−MS, alperujo, fusty/muddy sediment defect

## Abstract

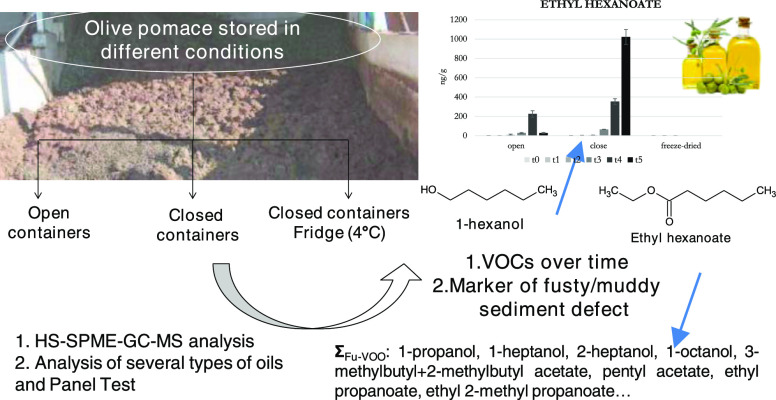

An olive pomace from the two-phase decanter stored in different conditions was used as a model to simulate the detrimental biological phenomena occurring during olive oil processing and storage. A group of EVOO and defective oils were also analyzed. The volatile fraction was studied with HS-SPME-GC–MS; 127 volatiles were identified (55 of which tentatively identified) and evaluated over time. Seven volatiles were tentatively identified for the first time in olive oil; the role of C6 alcohols in detrimental biological phenomena was highlighted. Suitable volatile markers for defects of microbiological origin were defined, particularly the fusty/muddy sediment. They were then applied to olive oils with different quality categories; one of the markers was able to discriminate among EVOOs and all the defective samples, including the borderline ones. The marker was constituted by the sum of concentrations of 10 esters, 4 alcohols, 1 ketone, and 1 α-hydroxy-ketone but no carboxylic acids.

## Introduction

Extra virgin olive oil (EVOO), the premium commercial subcategory of virgin olive oil (VOO), is the most valuable category of olive oil and is well-recognized as the vegetable oil with the highest sensory and nutraceutical quality. EVOO has a unique taste and aroma and a high content of oleic acid and is characterized by the presence of minor compounds such as volatile organic compounds (VOCs) and bioactive phenols.^1−7^ The commercial classification of VOO is based on legal limits of both chemical and sensory characteristics.^8,9^ As for the sensory assessment, the detection of specific positive and negative attributes is performed by the panel test. The panel test has contributed to improving the VOO quality in the last three decades, but it still presents some criticisms, such as slowness, sometimes low reproducibility, high costs, and mainly some difficulties in classification of the so-called borderline oils, particularly EVOO *vs* VOO.^8,10^ Since the attributes evaluated by the panel test are mainly ascribable to several VOCs, information about the relationship between VOCs and sensory attributes is very helpful in order to develop a reliable tool to support the panel test.^8,11,12^ Several VOCs generated by the C6 and C5 branches of the so-called lipoxygenase pathway (LOX pathway) have been identified as responsible for the positive green and fruity attributes.^13^ Similarly, the VOCs responsible for the rancid defect, triggered by fatty acid oxidation, are quite defined, and some markers for the rancid defect have been proposed over time.^5,8,14^ On the other hand, the sensory defects caused by biological phenomena, such as the fusty/muddy sediment, the musty/humid, and the winey-vinegary/acid-sour, have not yet been clearly related to specific VOCs due to several reasons. Several types of microorganisms (belonging to yeasts, molds, and bacteria) are responsible for the enzymatic activities causing the formation of several VOCs and, probably, leading to different nuances of the defect. The definition of some defects not always fully separable from each other hides different nuances and includes different biological phenomena. For example, the fusty/muddy sediment sensory attribute was inserted with the Reg. EC. 640/2008 by merging two quite different defects: (i) the fusty defect, which indicates the characteristic flavor of virgin olive oils from inadequately stored olives; (ii) the muddy sediment defect, which indicates the characteristic flavor of VOOs left in prolonged contact with water and solid particles during storage (i.e., the unfiltered oils). More generally, the different nuances of this defect can originate from several situations involving different kinds of substrates under physical structure conditions (e.g., late olive ripening, inadequate conservation of olives before oil processing, olive milling for oil production, storage of unfiltered oils).^1,15−20^

Several classes of VOCs have been reported in the literature as generated from detrimental biological activities, such as esters, carboxylic acids, hydrocarbons, ketones, alcohols, terpenes, volatile phenols, and short-branched aldehydes.^1,15,17,18,21−26^ Some of these VOCs were differently associated with specific off-flavors: butanoates and 2-ethyl butanoates were associated with the “muddy sediment” defects; *p*-cresol may contribute to the fecal off-flavor of muddy olive oils, and 4-ethylphenol is responsible for “horse sweat, barnyard, burnt plastic” off-flavors.^1,17,26^ The musty defect was linked with the C8 VOCs,^1^ but some disagreements have emerged in the literature.^5,8,20,21,23^ The appearance of the fusty defect was observed when the content of some VOCs derived from amino acid metabolism reached quite high values, but fusty oils in which these VOCs were absent were also found.^15^ More recently, a group of 10 VOCs have been identified in veiled VOOs assessed as fusty by the panel test.^25^ The winey-vinegary defect was related to the growth of yeasts causing aerobic fermentation, and it is associated with acetic acid, ethanol, and ethyl acetate.^8,13,20,27^

Extraction of virgin olive oil is increasingly carried out by the two-phase decanter, which produces as a by-product an olive pomace, often called “alperujo”. Alperujo can be discarded, used to recover added-value,^28−33^ or stored in open air for a long time and then used to produce the second centrifugation olive oil, named “remolido”.^34,35^ Very high amounts of 4-ethylphenol, likely formed by the activity of *Lactobacillus pentosus* through decarboxylation of *p*-coumaric and ferulic acids during long-term storage of olive pomace, were detected in remolido.^34,36^ Many differences have been observed between the composition of the volatile fraction of remolido in comparison with that of VOO.^35^

New approaches and systematic studies should be carried out for a more detailed definition of the VOCs responsible for the sensory defects caused by biological phenomena in order to establish reliable molecular markers as in the case of rancid^5^ and winey-vinegary defects.^27^ Since the alperujo appeared as an evolving system where several biological transformations occur and strongly affect the volatile profile, in this study, it was used as a model to stress the detrimental biological phenomena occurring during olive oil processing and storage. The volatile profile of alperujo was studied, and the VOCs that showed the largest differences over time were proposed for discriminating olive oils with the fusty/muddy sediment defect from EVOOs. A group of extra virgin or defective oils were analyzed to evaluate the suitability of the proposed group of VOCs.

## Material and Methods

### Chemicals and Standard Preparation

4-Methyl-2-pentanol (≥98.0%) used as the internal standard (ISTD) for semiquantitative analysis of VOCs and all the 73 external standards (Table S1) used for compound identification confirmation were from Sigma-Aldrich (Steinheim, Germany): their purity is given in Table S1. Solutions of the above VOCs were prepared using a refined olive oil free from VOCs. A linear alkane mixture (C9–C30) in hexane was also purchased from Sigma Aldrich (St. Louis, MO, USA).

### Samples

#### Olive Pomace/Alperujo

80 kg of alperujo sample, hereinafter two-phase olive pomace, was collected on November 14, 2019, in a farm located in San Casciano Val di Pesa (Florence, Italy) immediately after oil extraction by the two-phase decanter (Toscana Enologica Mori, Florence, Italy). The starting material was a batch of healthy olives constituted by a mix of the three typical Tuscan cultivars (i.e., approx. 40% Frantoio cv, 40% Moraiolo cv, and 20% Leccino cv).

#### Olive Oils

A set of 11 olive oils were selected as follows:

(a) four EVOOs from different Italian regions (the **EVOO1**, **EVOO2**, **EVOO3**, and **EVOO4** samples from Sicily, Calabria, Campania, and Apulia, respectively), collected during the 2019/2020 olive oil crop season during virgin olive oil competitions;
(b) one virgin olive oil (the **VOO** sample) and two lampante virgin olive oils (the **LVOO1** and **LVOO2** samples) with fusty/muddy sediment as the main sensory defect, collected during the 2019/2020 olive oil crop season from the Carapelli laboratory (Tavarnelle Val di Pesa, Florence, Italy);
(c) two IOC reference oil samples, one labeled for the fusty defect (the **Fusty-IOC** sample) and the other one labeled for the fusty/muddy sediment defect (the **FustyMuddy-IOC** sample). They were slightly diluted with a refined olive oil in order to have a median of the defect of approx. 6, thus classifiable as lampante virgin olive oils;
(d) one sample was the so-called “remolido” (the **Remolido** sample), collected during the 2019/2020 olive oil crop season from the Carapelli laboratory;
(e) one sample was collected from the residue precipitated in a tank containing an unfiltered VOO and was stored for 3 months in a bottle in contact with water and solid particles (the **Residue** sample) and was provided by the Carapelli laboratory during the 2019/2020 crop.

Once collected, oil samples were stored in the dark at room temperature until analysis.

### Experimental Design

The olive pomace sample arrived in the laboratory approx. 1 h after collection. An approx. 5 kg aliquot was immediately freeze-dried until reaching a constant weight; the freeze-dried pomace was split in three airtight containers and stored in the dark at room temperature (the **freeze-dried** alperujo samples). Other aliquots of the fresh olive pomace were put in 5 L containers and stored as follows: (i) three were hermetically sealed and stored at room temperature (ranging 20–23 °C) in order to simulate anaerobic operating conditions (the **closed** alperujo samples); (ii) three were kept open and stored at room temperature in order to simulate aerobic operating conditions (the **open** alperujo samples).

For VOC analysis, aliquots of the **open** samples were withdrawn on the sample surface and no mixing was applied; aliquots of the **freeze-dried** and **closed** samples were instead withdrawn in the sample bulk. Analysis of VOCs was performed at time 0 (i.e., approx. 2 h after olive milling) and after 1, 2, 4, 7, and 10 days for **open** and **closed** samples; for the **freeze-dried** samples, analyses were at time 0 and after 21 and after 45 days of storage.

### Olive Pomace Water and Oil Contents

The water content of the olive pomace sample was evaluated by lyophilizing a 1 kg aliquot until reaching a constant weight (3 days). The oil content was evaluated on the freeze-dried material by extraction with hexane, as previously reported.^37^ Both the measurements were performed in triplicate.

### Olive Oil Sample Characterization

Free fatty acids, peroxide number, and UV spectrophotometric indices (*K*_232_, *K*_268_, and Δ*K*) were evaluated according to the official analytical methods.^9^ For assessment of the sensory characteristics, samples were evaluated according to the same regulation^9^ by a panel acknowledged by the Italian Ministry of Agricultural Policies (MIPAAF) as previously described.^5^

### HS-SPME-GC–MS Analysis of Volatile Organic Compounds

For analysis of VOCs in olive oil and alperujo samples, an aliquot of 4.3 g of sample (only for **freeze-dried** alperujo samples it was 1.0 g) was weighed in a 20 mL screw cap vial and was spiked with 0.1 g of a solution of internal standard (4-methyl-2-pentanol, 10.2 μg/g in a refined olive oil previously analyzed and free from VOCs). After sample and internal standard addition, the vial was vigorously shaken for obtaining a mixture as homogeneous as possible. After sample equilibration for 5 min at 45 °C, a 1 cm 50/30 μm fiber coated with DVB/CAR/PDMS (Agilent, Palo Alto, CA, USA) was exposed to the vial headspace under orbital shaking (400 rpm) for 20 min, and the adsorbed VOCs were then desorbed for 1.7 min at 260 °C in the injection port of a 6890 N GC equipped with a model 5975 MS detector (Agilent, Palo Alto, CA, USA). After each analysis, a fiber backout of 20 min at 260 °C was performed in a backout unit. The VOCs were separated in a HP-Innowax capillary column (50 m × 0.2 mm id, 0.4 μm film thickness). The carrier gas was helium at 1.2 mL/min, and the oven temperature changed as follows: after 2 min at 40 °C, it was raised at 156 °C at 4 °C/min and then at 260 °C at 10 °C/min. Ion source and transfer line temperatures were 230 and 250 °C, respectively. The mass detector was set to work in full scan mode with a 70 eV ionization energy in the mass range of 29–350 Th, 1500 Th/s. A mixture constituted by C9-C30 linear alkanes in hexane was also analyzed in the same conditions of samples for calculation of retention indices of peaks.^38^

Identification of VOCs was performed as follows: if the commercial standard was available, peak identification was confirmed comparing its mass spectrum, retention time, and retention index with those of the standard. The other VOCs were tentatively identified by comparing the mass spectra of the peak with the mass spectra reported in the database of the standard NIST08/Wiley98 library (minimum matching factor, 80%) and comparing their retention indexes with those found in the NIST Standard Reference Database (Table 1 and Table S1).^38^

**Table 1:** VOC Data Processing of Open and Closed Alperujo Samples^a^

VOC	time	storage	storage × time	attribution	identification^b^
acetic acid	1.954 × 10^–29^	2.842 × 10^–24^	4.386 × 10^–26^		STD
3-methyl butanoic acid	1.731 × 10^–27^	3.475 × 10^–22^	1.749 × 10^–26^		tentative
(*E*)-2-pentenoic acid	6.445 × 10^–25^	3.673 × 10^–20^	2.536 × 10^–24^	not detected in olive oils	tentative
pentanoic acid	3.642 × 10^–24^	1.881 × 10^–19^	2.198 × 10^–22^		STD
propanoic acid	2.718 × 10^–22^	1.929 × 10^–18^	3.748 × 10^–21^		STD
methyl 3-methylbutanoate	2.771 × 10^–22^	2.274 × 10^–16^	1.836 × 10^–21^		tentative
2-methyl propanoic acid	4.117 × 10^–22^	4.737 × 10^–18^	2.935 × 10^–21^		tentative
acetoin acetate	6.607 × 10^–21^	7.281 × 10^–16^	6.607 × 10^–21^	not detected in olive oils	tentative
2-methyl butanoic acid	1.319 × 10^–20^	5.918 × 10^–15^	4.459 × 10^–19^		tentative
1-octen-3-ol	1.671 × 10^–20^	1.109 × 10^–15^	6.791 × 10^–20^		STD
methyl acetate	1.888 × 10^–20^	2.050 × 10^–13^	4.532 × 10^–17^		STD
3-pentanol	3.093 × 10^–20^	2.267 × 10^–14^	6.794 × 10^–20^		tentative
(*E*)-3-hexenoic acid	2.527 × 10^–19^	1.494 × 10^–14^	3.914 × 10^–17^		tentative
1-octen-3-one	1.632 × 10^–18^	2.733 × 10^–12^	1.387 × 10^–15^		STD
2-methyl-1-butanol + 3-methyl-1-butanol	2.257 × 10^–18^	6.358 × 10^–11^	3.100 × 10^–15^		STD
4-hepten-1-ol	6.187 × 10^–18^	5.386 × 10^–18^	1.323 × 10^–17^		tentative
ethyl benzoate	1.727 × 10^–17^	3.773 × 10^–13^	2.076 × 10^–16^		tentative
ethyl hexanoate	6.152 × 10^–17^	2.742 × 10^–12^	4.805 × 10^–16^		tentative
ethyl-(*Z*)-3-hexenoate	2.522 × 10^–16^	1.908 × 10^–07^	3.725 × 10^–17^	trace amount in oils	tentative
octanoic acid	4.330 × 10^–16^	4.557 × 10^–09^	1.615 × 10^–09^		tentative
hexanoic acid	7.365 × 10^–16^	8.858 × 10^–04^	1.986 × 10^–04^		STD
styrene	9.846 × 10^–16^	*0.451*	6.335 × 10^–06^		tentative
nonyl acetate	1.066 × 10^–15^	2.965 × 10^–08^	1.376 × 10^–10^		tentative
methyl hexanoate	1.674 × 10^–15^	4.823 × 10^–09^	3.125 × 10^–04^		tentative
2-phenylethanol	2.315 × 10^–15^	1.501 × 10^–05^	1.248 × 10^–12^		STD
butanoic acid	2.523 × 10^–15^	5.280 × 10^–08^	1.743 × 10^–08^		STD
heptanoic acid	6.799 × 10^–15^	3.187 × 10^–09^	9.220 × 10^–13^		tentative
1-heptanol	9.427 × 10^–15^	1.685 × 10^–15^	5.569 × 10^–14^		STD
1-propanol	9.645 × 10^–15^	1.355 × 10^–11^	3.757 × 10^–14^		STD
hexyl acetate	1.344 × 10^–14^	3.414 × 10^–05^	4.811 × 10^–13^	LOX*	STD
ethyl acetate	1.576 × 10^–14^	*0.357*	2.599 × 10^–12^		STD
2-methyl-1-propanol	2.218 × 10^–14^	1.372 × 10^–05^	4.197 × 10^–13^		STD
ethyl 2-methylpropanoate	6.842 × 10^–14^	4.250 × 10^–05^	3.410 × 10^–07^		tentative
pentyl acetate	1.686 × 10^–13^	8.180 × 10^–04^	1.092 × 10^–11^		tentative
1-nonanol	1.906 × 10^–13^	9.019 × 10^–09^	3.287 × 10^–09^		STD
1-decanol	2.170 × 10^–13^	4.529 × 10^–12^	2.618 × 10^–11^	not detected in olive oils	tentative
(*Z*)-2-pentenyl acetate	3.354 × 10^–13^	9.829 × 10^–08^	2.222 × 10^–10^	not detected in olive oils	tentative
2-methyl-2,3-pentanediol	6.607 × 10^–13^	1.446 × 10^–11^	9.861 × 10^–12^	trace amount in oils	tentative
ethyl 3-methylbutanoate	1.437 × 10^–12^	2.491 × 10^–04^	4.167 × 10^–04^		tentative
2-methylbutyl acetate + 3-methylbutyl acetate	4.326 × 10^–12^	*0.922*	1.556 × 10^–06^		tentative
2-hydroxy-3-pentanone	7.158 × 10^–12^	1.433 × 10^–09^	1.668 × 10^–10^	trace amount in oils	tentative
octane	1.242 × 10^–11^	1.362 × 10^–06^	0.005		STD
ethyl nonanoate	1.261 × 10^–11^	1.055 × 10^–07^	1.425 × 10^–08^	not detected in olive oils	tentative
ethyl octanoate	1.503 × 10^–11^	2.853 × 10^–09^	1.532 × 10^–10^		tentative
(*E*)-2-hexen-1-ol	1.719 × 10^–11^	*0.025*	*0.019*	LOX*	STD
(*E,E*)-2,4-hexadienal	1.739 × 10^–11^	*0.224*	*0.689*	no increase in alperujo	STD
2,2-dimethyl-1-propanol	2.806 × 10^–11^	1.002 × 10^–10^	2.556 × 10^–08^	not detected in olive oils	tentative
(*Z*)-3-hexenyl acetate	3.456 × 10^–11^	*0.272*	7.235 × 10^–10^	LOX	STD
ethyl decanoate	6.567 × 10^–11^	2.970 × 10^–08^	3.845 × 10^–10^	not detected in olive oils	tentative
methyl octanoate	7.854 × 10^–11^	7.674 × 10^–12^	5.876 × 10^–10^	not detected in olive oils	tentative
methyl propanoate	1.357 × 10^–10^	1.375 × 10^–05^	5.065 × 10^–05^		STD
2,2-dimethyl propanoic acid	1.539 × 10^–10^	1.682 × 10^–06^	2.669 × 10^–09^	Trace amount in oils	tentative
1-butanol	1.999 × 10^–10^	3.692 × 10^–07^	3.089 × 10^–09^		tentative
benzyl alcohol	2.552 × 10^–10^	1.812 × 10^–05^	2.267 × 10^–09^		tentative
3-hydroxy-2-butanone (acetoin)	2.991 × 10^–10^	1.032 × 10^–08^	1.608 × 10^–10^		tentative
heptyl acetate	3.266 × 10^–10^	*0.258*	0.027	not detected in olive oils	tentative
(*E*)-2-hexenal	5.665 × 10^–10^	*0.199*	*0.600*	LOX	STD
ethyl butanoate	1.260 × 10^–09^	8.755 × 10^–04^	4.689 × 10^–08^		STD
(*E*)-2-penten-1-ol	1.305 × 10^–09^	0.016	*0.117*	LOX	STD
(*Z*)-3-hexenal	1.487 × 10^–09^	*0.292*	*0.755*	LOX	STD
limonene	1.514 × 10^–09^	*0.136*	*0.233*	no increase in alperujo	STD
ethyl 3-methyl-2-butenoate	1.756 × 10^–09^	*0.117*	0.461	not detected in olive oils	tentative
isobutyl acetate	2.137 × 10^–09^	*0.155*	6.119 × 10^–07^		tentative
ethyl propanoate	2.188 × 10^–09^	0.024	1.310 × 10^–07^		STD
ethyl heptanoate	2.296 × 10^–09^	0.002	3.551 × 10^–05^		tentative
2-octanone	3.187 × 10^–09^	*0.363*	*0.956*		STD
ethanol	5.475 × 10^–09^	1.579 × 10^–05^	6.683 × 10^–08^		STD
1-hexanol	5.951 × 10^–09^	4.961 × 10^–08^	2.911 × 10^–08^	LOX*	STD
1-pentanol	7.227 × 10^–09^	1.042 × 10^–09^	7.636 × 10^–09^		STD
methyl decanoate	3.375 × 10^–08^	4.390 × 10^–08^	2.424 × 10^–07^	not detected in olive oils	tentative
1-hydroxy-2-propanone	5.775 × 10^–08^	3.565 × 10^–06^	2.269 × 10^–08^	not detected in olive oils	tentative
(*E*)-2-pentenal	6.702 × 10^–08^	*0.057*	*0.582*	LOX	STD
1-penten-3-one	7.523 × 10^–08^	*0.161*	*0.454*	LOX	STD
methyl heptanoate	9.599 × 10^–08^	0.001	0.003	no increase in alperujo	tentative
2,2-dimethyl-1-propyl acetate	9.810 × 10^–08^	2.275 × 10^–06^	9.810 × 10^–08^	not detected in olive oils	tentative
1-octanol	1.105 × 10^–07^	7.890 × 10^–08^	7.789 × 10^–07^		STD
nonanoic acid	3.805 × 10^–07^	7.086 × 10^–06^	1.854 × 10^–06^		tentative
2,3-butanedione	7.264 × 10^–07^	5.510 × 10^–05^	0.002	trace amount in oils	tentative
4-ethyl-phenol	7.279 × 10^–07^	4.639 × 10^–05^	7.279 × 10^–07^		STD
2-pentanol	2.209 × 10^–06^	*0.753*	0.001		STD
ethyl pentanoate	5.990 × 10^–06^	*0.488*	7.723 × 10^–06^		tentative
(*E*)-3-hexen-1-ol	8.747 × 10^–06^	1.862 × 10^–12^	3.832 × 10^–08^		STD
(*Z*)-3-hexen-1-ol	1.318 × 10^–05^	5.172 × 10^–05^	1.020 × 10^–06^	LOX	STD
2-heptanol	1.838 × 10^–05^	*0.054*	*0.172*		STD
(*Z*)-2-penten-1-ol	4.421 × 10^–05^	5.874 × 10^–05^	0.028	LOX	STD
(*Z*)-2-hexen-1-ol	7.967 × 10^–05^	5.631 × 10^–12^	6.564 × 10^–05^		STD
3-methylbutanal	9.041 × 10^–05^	*0.092*	0.036	no increase in alperujo	STD
(*E*)-2-heptenal	1.260 × 10^–04^	0.033	*0.262*	no increase in alperujo	STD
heptanal	1.331 × 10^–04^	*0.109*	*0.114*	no increase in alperujo	STD
hexanal	1.591 × 10^–04^	0.009	0.039	no increase in alperujo - LOX	STD
2-methylbutanal	3.968 × 10^–04^	*0.110*	*0.080*	no increase in alperujo	STD
2-methylpropanal	7.785 × 10^–04^	*0.209*	*0.249*	no increase in alperujo	tentative
(*E*,*E*)-2,4-nonadienal	0.001	*0.061*	*0.242*	*p* > 0.001	STD
1-penten-3-ol	0.001	0.007	*0.740*	*p* > 0.001	STD
(*e*)-2-octenal	0.001	0.012	*0.344*	*p* > 0.001	STD
2-heptanone	0.001	*0.928*	0.035	*p* > 0.001	STD
acetaldehyde	0.002	0.004	2.478 × 10^–07^	*p* > 0.001	tentative
pentanal	0.002	*0.419*	0.041	*p* > 0.001	STD
(*E*,*E*)-2,4-heptadienal	0.004	9.060 × 10^–04^	*0.102*	*p* > 0.001	STD
methyl isobutyl ketone	0.004	0.007	0.003	*p* > 0.001	tentative
(*E*)-2-decenal	0.004	0.032	*0.287*	*p* > 0.001	STD
octanal	0.005	0.002	*0.130*	*p* > 0.001	STD
methanol	0.007	1.350 × 10^–06^	0.023	*p* > 0.001	STD
4-hexen-2-one	0.010	2.023 × 10^–08^	0.002	*p* > 0.001	tentative
nonanal	*0.079*	0.048	*0.602*	*p* > 0.001	STD
(*E*,*E*)-2,4-decadienal	*0.083*	0.003	*0.511*	*p* > 0.001	STD
methyl nonanoate	*0.115*	3.709 × 10^–05^	0.024	*p* > 0.001	tentative
butanone	*0.168*	*0.682*	0.004	*p* > 0.001	STD
benzaldehyde	*0.234*	4.838 × 10^–07^	2.386 × 10^–07^	*p* > 0.001	STD
6-methyl-5-hepten-2-one	*0.307*	*0.290*	*0.579*	*p* > 0.001	STD
3-pentanone	*0.401*	0.011	3.971 × 10^–05^	*p* > 0.001	STD
heptane	*0.428*	*0.093*	*0.376*	*p* > 0.001	STD
toluene	*0.434*	*0.185*	*0.372*	*p* > 0.001	tentative

a *p* values calculated for each VOC by two-factor ANOVA, where the two factors were the storage time (time) and the type of storage (storage). The two-way interaction time × storage is also reported. Nonsignificant values (*p* > 0.05) are in italic. The approach for identification of each VOC is reported in the last column.

b Identification: “STD” means that identification was confirmed with the mass spectrum and retention index in accordance with the commercial standard; “tentative” means that the molecule was tentatively identified matching the mass spectrum with the NIST08/Wiley98 library and the retention index with the NIST Chemistry WebBook

#### The Semiquantitative Approach

An accurate quantitative approach, for example, by preparing calibration curves in model solution or using the method of standard addition,^39^ was not possible at this level of research. In fact, the analysis was partially untargeted (i.e., we did not know what VOCs were present in the pomace stored in different conditions over time, and a total of approx. 130 VOCs in very different amounts were indeed detected, some of them tentatively identified). However, for the aim of this study, it was important to follow the evolution of VOCs over time rather than their precise absolute quantitation in each sample. Bearing this in mind and trying to quantitate each VOC in a way as accurate as possible, we adopted the following approach. For each VOC, quantifier and qualifier ions were selected (Table S1) allowing for a complete separation from other co-eluting VOCs: when the base peak (i.e., intensity of the ion, 100%) permitted the complete peak separation, it was selected as the quantifier; in other cases, the quantifier was selected as the most intense peak in the mass spectrum that permitted resolution of peaks that co-eluted in scan mode. For each compound, peak area was obtained from the extract ion chromatogram (EIC) using the quantifier, and semiquantitation was carried out after area normalization with 4-methyl-2-pentanol as the internal standard. For the VOCs quantitated using a fragment with abundance less than 50% of the base peak as the quantifier, the normalization with the ISTD was carried out using the area of a minor fragment of the ISTD spectra (i.e., the ion at *m*/*z* = 45 Th, instead of that of the base peak at 69 Th). Concentration of each VOC was consequently calculated according to the following equation
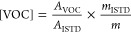
where [VOC] is the VOC concentration in μg/g, *A*_VOC_ is the peak area of the quantifier ion for that VOC, *A*_ISTD_ is the peak area of the ISTD (ion 45 if the quantifier of the quantitated VOC is greater than 50% of the base peak; ion 69 if it is lower than 50% of the base peak), *m*_ISTD_ (μg) is the mass of the ISTD added to the sample, and *m* is the sample weight (g).

### Statistical Analysis

The data are from three independent samples and are reported as mean ± SEM. For each analyzed VOC in alperujo, two-factor ANOVA was run for assessing the effect or type of storage, storage time, and their interaction, and the obtained results are presented in Table 1, showing the *p*-values. All statistical analyses were performed using Microsoft Excel Statistical Software with additional tools provided by the software DSAASTAT v1.1.^40^

## Results and Discussion

### Characterization of Alperujo Samples

The water content of the alperujo sample was 75.3%, and the oil content was 11% on a dry matter basis (i.e., 2.8% on a total content basis). Analysis of the volatile profile of alperujo samples during storage pointed out the presence of a total of 127 VOCs (55 of which tentatively identified, Table S1), among which 35 esters, 32 alcohols (one is a diol), 22 aldehydes, 14 ketones (one of which is α-diketone and three α-hydroxy ketones), 14 carboxylic acids, 5 hydrocarbons (2 aromatic, 2 aliphatic, and 1 monoterpene), 4 volatile phenols, and 1 furan. Fourteen VOCs were detected in trace amounts in alperujo samples. Since their contents did not significantly change over time in none of the storage conditions, these 14 VOCs (indicated with “*” in Table S1) were excluded from the statistical data processing. The significance of the effect of storage conditions, storage time, and their interaction on the variation of the selected 113 VOCs is reported in Table 1.

The alperujo samples were treated in order to have the following different susceptibilities to biological phenomena: (i) the open alperujo samples were susceptible to microbial activities in aerobic conditions since they had a high level of water content and oxygen exposure; (ii) the closed alperujo samples were susceptible to microbial activities in anaerobic conditions since they had a high level of water content and no oxygen exposure; (iii) the freeze-dried alperujo samples were not susceptible to biological phenomena since they had no water content. Therefore, the significant increase of the VOC content during storage in the open and closed alperujo samples can be related to the metabolism of the spoilage microorganisms, being the freeze-dried ones the reference samples where any biological phenomena cannot occur.

**Figure 1:**
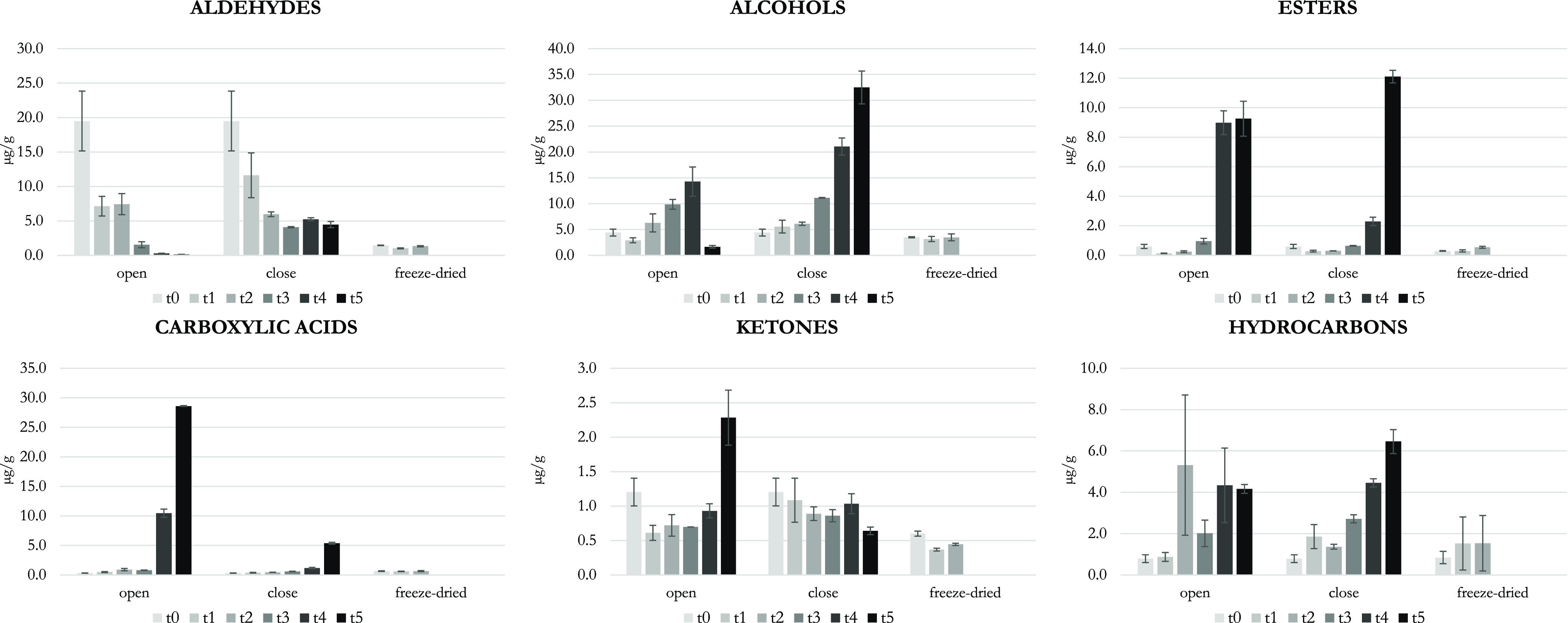
Evolution of the sum of aldehydes, alcohols, esters, carboxylic acids, ketones, and hydrocarbons in alperujo samples. Data are expressed on a dry matter basis. Standard error of the mean (SEM) is the sum of the SEMs of each VOC in a class. t0, 0 days; for open and closed samples t1, 1 day; t2, 2 days; t3, 4 days; t4, 7 days; t5, 10 days; for the freeze-dried sample, t1, 21 days; t2, 45 days.

**Figure 2:**
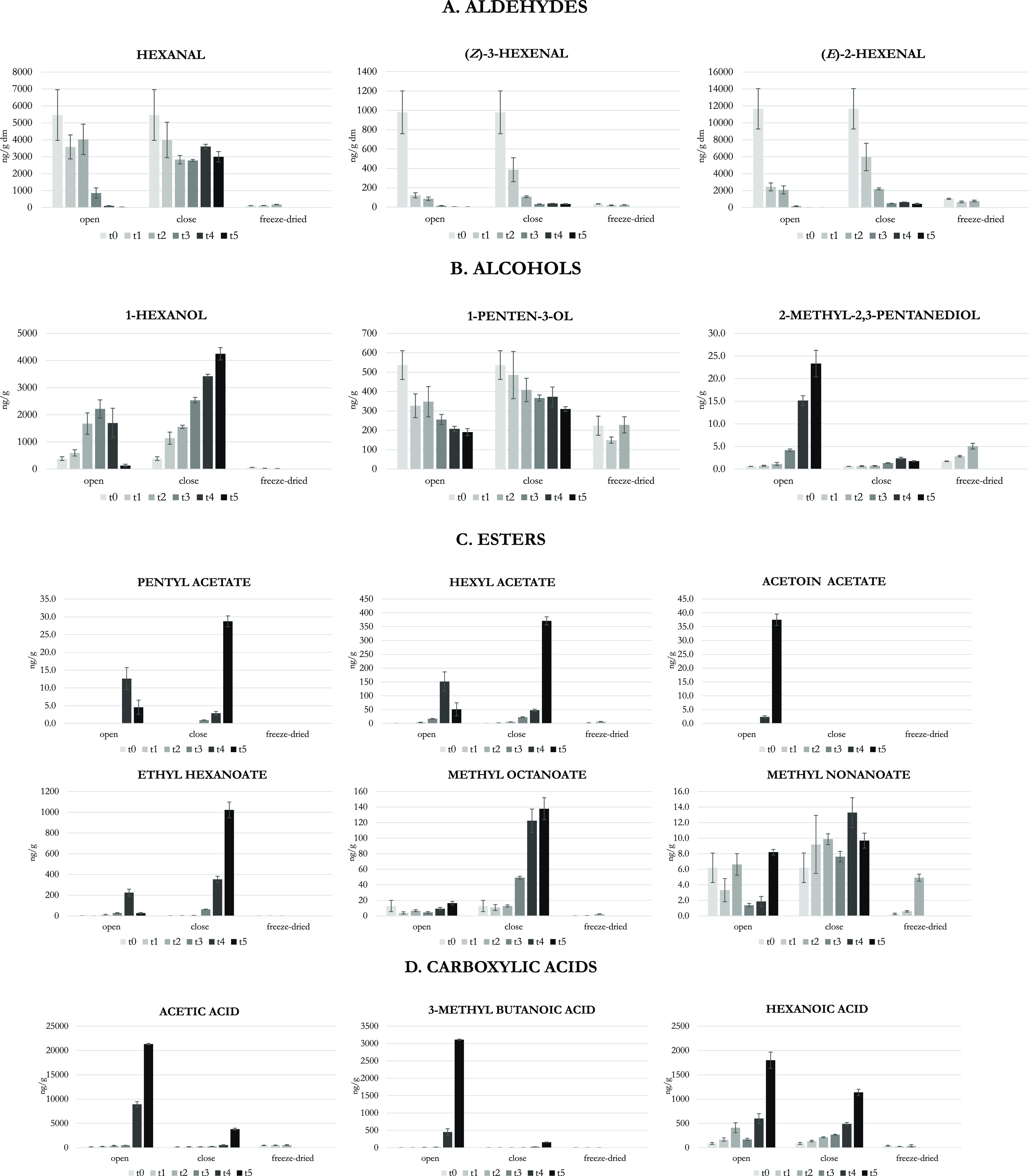
Evolution over time of selected aldehydes (A), alcohols (B), esters (C), and carboxylic acids (D) in alperujo samples. Data are the mean of three independent determinations and are expressed on a dry matter basis, and the standard error of the mean is also reported in the charts. t0, 0 days; for open and closed samples t1, 1 day; t2, 2 days; t3, 4 days; t4, 7 days; t5, 10 days; for the freeze-dried sample, t1, 21 days; t2, 45 days.

Figure 1 shows the evolution of the sum of VOCs belonging to different classes, namely, aldehydes, alcohols, esters, carboxylic acids, ketones, and hydrocarbons (the sum of volatile phenols was not reported due to the quite low amount) in different storage conditions. Examples of the evolution of specific VOCs are given in Figure 2.

The aldehydes were not related to the biological phenomena. The total content of aldehydes was much higher in the open and closed samples than in the freeze-dried sample at time 0 due to the stripping of the volatile compounds under vacuum conditions during the freeze-drying treatment.^16^ It significantly decreased in both open and closed samples during storage (Figure 1). In the open and closed alperujo samples at time 0, the LOX aldehydes (*E*)-2-hexenal (≈12,000 ng/g dm), hexanal (≈5000 ng/g dm), and (*Z*)-3-hexenal (≈1000 ng/g dm) were the aldehydes present in highest amounts (Figure 2A), and they almost disappeared at the end of the experiment with the only exception given by hexanal in the closed sample (whose content halved). The contents of almost all aldehydes significantly decreased over time in the open and closed alperujo samples, with exceptions given by acetaldehyde and benzaldehyde, which showed a not clear trend (data not shown). In the freeze-dried samples, the contents of some aldehydes showed an opposite trend (Supporting information, Figure S1), with an increase of pentanal, nonanal, (*E*)-2-heptenal, hexanal, heptanal, octanal, (*E*,*E*)-2,4-heptadienal, and (*E*,*E*)-2,4-nonadienal, aldehydes previously linked to fatty acid oxidation.^5^ This phenomenon, suggesting that the oil remained in the dried alperujo is exposed to chemical modifications, particularly oxidation, presumably due to the low levels of water activity in the dried alperujo, indicates that the freeze-dried alperujo is more susceptible to oxidation than the wet alperujo.

The alcohols were related to the biological phenomena, and their formation due to microbial activities in anaerobic conditions seemed to prevail during alperujo storage. The total content of alcohols was quite similar in all alperujo samples at time 0 (Figure 1), and it showed a significant increase, particularly after 4 days, only in the open and closed samples. Methanol, ethanol, and the LOX alcohols, namely, 1-penten-3-ol, (*Z*)-2-penten-1-ol, 1-hexanol, (*Z*)-3-hexen-1-ol, and (*E*)-2-hexen-1-ol, were present in high amounts (i.e., greater than 400 ng/g) in the open and closed samples at time 0, while all other alcohols were absent or present in amounts lower than 80 ng/g. In the freeze-dried samples, methanol (≈1300 ng/g), ethanol (≈1500 ng/g), 1-penten-3-ol (≈200 ng/g), and (*Z*)-2-penten-1-ol, and (*E*)-2-hexen-1-ol (≈100 ng/g) were the only alcohols present in significant amounts. The only four alcohols that decreased over time in both open and closed samples were 4 of the 6 LOX alcohols, namely, 1-penten-3-ol (Figure 2B), (*Z*)-2-penten-1-ol, (*E*)-2-penten-1-ol, and (*E*)-2-hexen-1-ol, whereas methanol only decreased in the open samples, likely due to its volatility. All other 23 alcohols increased during storage; 2,2-dimethyl propanol, 2-methyl-2,3-pentanediol (Figure 2B), and 1-octen-3-ol increased more in the open samples, whereas all the other 20 ones increased more in the closed samples. Ethanol (up to 19,000 ng/g), 1-hexanol (4000 ng/g), 2 + 3 methyl-1-butanol (3400 ng/g), and 2-phenylethanol (1700 ng/g) reached very high amounts at the end of storage. Concerning the behavior of 1-hexanol (Figure 2B), opposite to the other LOX-alcohols, its quick increase during storage was in agreement with data reported by other authors, which indicated this VOC also as a marker of fruit damage and microbial spoilage.^21,25,41,42^

The esters were related to the biological phenomena, and their formation seemed to be due to microbial activities in both aerobic and anaerobic conditions during alperujo storage. The total content of esters was quite low in both fresh and freeze-dried alperujo at time 0. It showed a sudden increase after 7 and 10 days in the open and closed samples, and only a slight increase in the freeze-dried one (Figure 1). The acetates represented 14 out of the 35 esters. With the only exception of butyl acetate and (*E*)-2-hexenyl acetate, the acetates showed an increase over time: overall in the open alperujo samples, the increase was particularly evident after 7 days. Then, some acetates continued to quickly increase (e.g., acetoin acetate, Figure 2C), while other ones (i.e., ethyl acetate, isobutyl acetate, pentyl acetate, hexyl acetate, (*Z*)-3-hexenyl acetate) decreased at the 10th day. In the closed alperujo samples, 2,2-dimethyl 1-propyl acetate, (*Z*)-2-pentenyl acetate, and acetoin acetate were absent, while all the other acetates showed a slow increase in the first 7 days of storage and a much faster increase in the following days (e.g., pentyl acetate and hexyl acetate, Figure 2C). Only methyl, ethyl, (*Z*)-2-pentenyl, and hexyl acetates were present in the freeze-dried alperujo samples, in amounts much lower than in the open and closed samples. Among the 21 esters other than acetates, only ethyl tiglate, methyl heptanoate, and methyl nonanoate did not show a clear trend over time in the open and closed alperujo samples, whereas ethyl 3-methylbut-2-enoate decreased over time and all the other ones increased over time in both storage conditions. The above behavior was different with respect to the acetates; none of them reached high amounts in the open alperujo samples, and many of them (particularly, all the methyl and ethyl esters of acids with an even number of C) increased in a faster way in closed than in open alperujo samples (Figure 2C). Almost all the above esters were absent in the freeze-dried alperujo sample, with the exception of methyl esters, which showed low but increasingly contents over time.

The carboxylic acids were related to the biological phenomena, and their formation due to microbial activities in aerobic conditions seemed to prevail during alperujo storage. The carboxylic acids were absent or present in quite low amounts (e.g., acetic acid, 174 ng/g; hexanoic acid, 95 ng/g) in the open and closed alperujo samples at time 0. The amounts of all the 14 acids strongly increased in the open samples over time, particularly after 7 days (Figure 1); octanoic acid was the only one that increased more in the closed than in open samples. In the open samples, acetic acid reached a very high amount (≈21,000 ng/g) followed by 3-methyl butanoic acid (≈3000 ng/g), hexanoic acid (≈2000 ng/g), 2-methyl propanoic acid (≈1000 ng/g), and 2-methyl butanoic acid (≈1000 ng/g), whereas in the closed samples, only acetic acid (≈4000 ng/g) and hexanoic acid (≈1000 ng/g) reached quite high amounts (Figure 2D). The strong increase of the contents of carboxylic acids in open containers was in agreement with previous papers, which reported an increase of acidity and volatile acids when alperujo was stored in open air ponds.^43,44^ In the freeze-dried alperujo samples, all acids were absent or present in negligible amounts, and none of them significantly increased over time.

The ketones were related to the biological phenomena, and their formation due to microbial activities in aerobic conditions seemed to prevail during alperujo storage. The total content of ketones was higher in open and closed samples than in the freeze-dried sample at time 0. No significant increases were observed in the freeze-dried samples during storage (Figure 1). 1-Penten-3-one (≈700 ng/g), 3-pentanone (≈300 ng/g), and 4-hexen-2-one (≈250 ng/g) were the ketones more representative in the open and closed samples at time 0, and 1-penten-3-one decreased over time in all samples. The ketones showed less evident increases over time than other VOCs. However, methyl isobutyl ketone showed the fastest increase in the open sample, and the C8 ketones, namely, 2-octanone and 1-octen-3-one, showed significant increases, particularly in the open samples. All the three α-hydroxy ketones, almost absent at time 0, quickly increased in the open samples, with 3-hydroxy-2-butanone (also known as acetoin) reaching 800 ng/g, whereas they showed only negligible variations in the closed samples.

The total hydrocarbons were related to the biological phenomena, and their formation seemed to be due to microbial activities both in aerobic and anaerobic conditions during alperujo storage. The total hydrocarbon content was similar in all samples at time 0, and then it showed an increase only in the open and closed samples, particularly after 4 days (Figure 1). Octane (≈500 ng/g at time 0) increased in the open sample (up to 3000 ng/g), but particularly in closed samples (up to 4500 ng/g); it was almost absent in the freeze-dried sample. Styrene, almost absent in all samples at time 0, strongly increased in both open (up to 800 ng/g) and closed (up to 1400 ng/g) samples. 4-Ethyl phenol was also detected in low but increasing amounts only in the open samples; high amounts of this compound were evidenced in lampante oils and particularly in remolido oils.^34^

### Characterization of Olive Oil Samples

The olive oil samples were chosen in order to have defects due to biological degradation changing as follows: (i) from olive oil samples with no defects (i.e., the EVOO samples) to olive oil samples with high intensity of defects (i.e., the LVOO samples); (ii) olive oil samples with high intensity of defects but with defects of different origin (i.e, the Remolido, the Residue, the fusty-IOC, and fusty/muddy sediment-IOC samples).

The four EVOO samples were all with free acidity below 0.25%, peroxide number below 10 m_eqO2_/kg, and spectrophotometric indices largely below the EVOO limits (i.e., *K*_232_, 2.50; *K*_270_, 0.22; Δ*K*, 0.010); they showed no presence of sensory defects, whereas the median of fruity was 7.7 for EVOO1, 7.6 for EVOO2, 6.8 for EVOO3, and 6.3 for EVOO4. The VOO sample was characterized by the presence of fruity notes with a median of 3.2 and the fusty/muddy sediment defect with a median of 1.7; free acidity was 0.18%, the peroxide number was 6.0 m_eqO2_/kg, and *K*_232_, *K*_270_, and Δ*K* were 1.51, 0.11, and 0.006, respectively. The LVOO1 and LVOO2 samples were characterized by free acidity higher than 1.5% and the presence of negligible fruity notes; the fusty/muddy sediment was the prevalent sensory defect with medians of 4.8 and 5.0, respectively. As stated in the Materials and Methods section, the two analyzed IOC reference oils (fusty-IOC and fusty/muddy sediment-IOC) showed a median of defect of 6. The Remolido and the Residue samples showed free acidity higher than 1.0%, a peroxide number higher than 20.0 m_eqO2_/kg, and negligible fruity notes and fusty/muddy sediment as the prevalent defects (medians of 4.4 and 5.2, respectively) followed by the presence of the winey-vinegary defect with a median of 1. In all the above defective samples, the rancid defect was absent or present with quite low intensity if compared with the fusty/muddy sediment defect.

Table 2 shows the contents of those olive oil VOCs commonly linked to the LOX pathway^13^ and to well-defined sensory defects such as rancid^5^ and winey-vinegary.^27^ The remaining VOCs of olive oil samples will be discussed in the next paragraph.

**Table 2:** Total Contents of the LOX-Related VOCs (Σ LOX) and of the Molecules Related to Rancid (Σ Rancid) and Winey/Vinegary (Σ Winey) Sensory Defects in the Olive Oil Samples^a^

sample	Σ LOX (μg/g)	Σ LOX ketones^b^ (%)	Σ LOX aldehydes^c^ (%)	Σ LOX esters^d^ (%)	Σ LOX C5 alcohols^e^ (%)	Σ LOX C6 alcohols^f^ (%)	Σ Rancid^g^ (μg/g)	Σ Winey^h^ (μg/g)
EVOO1	10.415	24.7%	48.3%	9.2%	9.5%	8.4%	0.051	0.375
EVOO2	11.806	25.2%	45.0%	12.0%	10.6%	7.2%	0.054	0.475
EVOO3	11.943	16.0%	59.3%	8.5%	9.3%	7.0%	0.039	0.645
EVOO4	17.131	11.4%	75.7%	1.1%	8.2%	3.6%	0.041	0.314
VOO	2.647	9.2%	23.7%	51.5%	4.9%	10.7%	0.032	0.876
LVOO1	3.431	0.4%	8.6%	6.1%	9.1%	75.8%	0.288	0.511
LVOO2	3.418	0.5%	9.9%	6.4%	9.1%	74.2%	0.341	0.536
FustyMuddyCOI	1.141	0.8%	83.9%	8.6%	5.5%	1.2%	0.206	0.393
FustyCOI	1.369	0.5%	62.3%	4.7%	2.2%	30.3%	0.302	0.089
Remolido	0.130	2.0%	33.6%	17.6%	8.2%	38.5%	0.040	1.216
Residue	6.792	0.4%	4.2%	6.6%	7.9%	80.9%	0.093	7.407

a The table also shows the percentage of each class of LOX-related VOCs. Data are expressed in μg/g for Σ LOX, Σ Rancid, and Σ Winey and in % for each class of LOX-related VOCs.

b 1-Penten-3-one.

c (*E*)-2-Pentenal, hexanal, (*Z*)-3-hexenal, (*E*)-2-hexenal.

d Hexyl acetate, (*Z*)-3-hexenyl acetate.

e (*E*)-2-Penten-1-ol, (*Z*)-2-penten-1-ol, 1-penten-3-ol.

f 1-Hexanol, (*E*)-3-hexen-1-ol, (*E*)-2-hexen-1-ol.

g Pentanal, nonanal, (*E*)-2-heptenal.

h Ethanol, ethyl acetate, acetic acid.

The content of LOX VOCs was higher in the EVOO samples than in the other samples, as expected (Table 2). In the 4 EVOOs, the aldehydes were the prevalent LOX VOCs (45.0–75.7%) followed by the ketones (namely, 1-penten-3-one, 11.4–25.2%); the C5 alcohols (8.2–10.6%), the esters (1.1–12.0%), and the C6 alcohols (3.6–8.4%) were present in lower percentages. Among the non-EVOO samples, the Residue sample showed the highest content of LOX-VOCs, followed by the two LVOO samples, but with a LOX-VOC profile very different from that of the EVOOs. In these samples, a clear prevalence of C6 alcohols, a low content of aldehydes (4.2–8.9%), and a negligible content of 1-penten-3-one were pointed out. In particular, it is interesting to note that the contents of 1-hexanol, (*E*)-2-hexen-1-ol, and hexyl acetate were detected in concentrations of 1 order of magnitude greater in the Residue than in EVOO samples. The higher contents of the C6 LOX-alcohols (*E*)-2-hexen-1-ol and 1-hexanol and the related acetates in non-EVOO than in EVOO samples are shown in Figure S2. This behavior, as already stated in the previous paragraph, is in agreement with data reported by other authors, which indicated the increase of C6 alcohols as a marker of fruit damage and microbial spoilage.^21,25,41,42^

Data concerning the VOCs linked to the rancid and winey-vinegary sensory defects were in good agreement with the sensory data from the panel test. As for the rancid defect, even if in this study data are only semiquantitative, all samples showed a content quite lower than the proposed limit for rancidity in olive oil (i.e., 0.65 μg/g);^5^ however, the LVOO and IOC reference oil samples showed a content 1 order of magnitude higher than the EVOO samples, suggesting that defective olive oil samples, even if not perceived as rancid, are usually more oxidized than EVOOs. As for the winey-vinegary defect, only the Remolido and Residue samples showed total contents of ethanol, ethyl acetate, and acetic acid significantly higher than all the other samples; in particular, in the Residue sample, the content was 1 order of magnitude higher than those in the other samples (Table 2). Despite the quite different content of the above VOCs in the Remolido and Residue samples, the perceived intensity of the winey-vinegary defect by the panel test was similar, likely due to the prevalence of the fusty/muddy sediment sensory defect.

### Markers for Sensory Defects Caused by Biological Phenomena

The 127 molecules identified or tentatively identified in the alperujo samples (Table S1) were compared with those found in the literature during an extensive bibliography research focused on the VOCs present in the virgin olive oil volatile fraction.^45^ Eleven out of the 127 VOCs (i.e., 2,3-butanedione, 2,2-dimethyl-1-propyl acetate, 2,2-dimethyl-1-propanol, ethyl 3-methylbut-2-enoate, ethyl (*Z*)-3-hexenoate, 2-methyl-2,3-pentanediol, 2-hydroxy-3-pentanone, acetoin acetate, (*E*)-4-hexen-1-ol, 4-hepten-1-ol, and 4-hexen-2-one) have never been identified in the volatile fraction of virgin olive oils so far. Searching for these molecules in the analyzed olive oil samples, four VOCs (i.e., 2,2-dimethyl-1-propyl acetate, 2,2-dimethyl-1-propanol, ethyl 3-methylbut-2-enoate, and acetoin acetate) were not detected in any sample. The other seven VOCs were instead detected for the first time in at least one defective sample, with only one of them (i.e., 4-hexen-2-one) detected in the EVOO samples. These preliminarily findings confirmed that the proposed approach is suitable to gain new useful information about the volatile fraction of virgin olive oils with defects originated by detrimental enzymatic activities of microorganisms and encouraged us to proceed in searching for volatile molecular markers of defects of microbiological origin capable of discriminating defective samples from EVOO samples.

A group of VOCs as potential markers were selected after excluding several VOCs on the basis of the following criteria: (i) the VOCs not showing a clear increasing trend in at least one among open and closed alperujo samples were excluded; (ii) the VOCs related to the LOX pathway (“LOX” in column “attribution” of Table 1) were excluded, with the exception of 1-hexanol, hexyl acetate, and (*E*)-2-hexen-1-ol (“LOX*”); (iii) the VOCs not detected in any of the analyzed olive oil samples (“not detected in olive oils” in column “attribution” of Table 1) or detected only in trace amounts (“trace amount in oils” in column “attribution” of Table 1) were excluded. Concerning point (i), a *p*-value higher than 1.00 × 10^–03^ for the effect of storage time (Table 1) was selected as the first exclusion criterion (“*p* > 0.001” in column “attribution” of Table 1); then, the VOCs not showing an increasing trend (“no increase in alperujo” in column “attribution” of Table 1) were also excluded, no matter their *p*-value.

**Figure 3:**
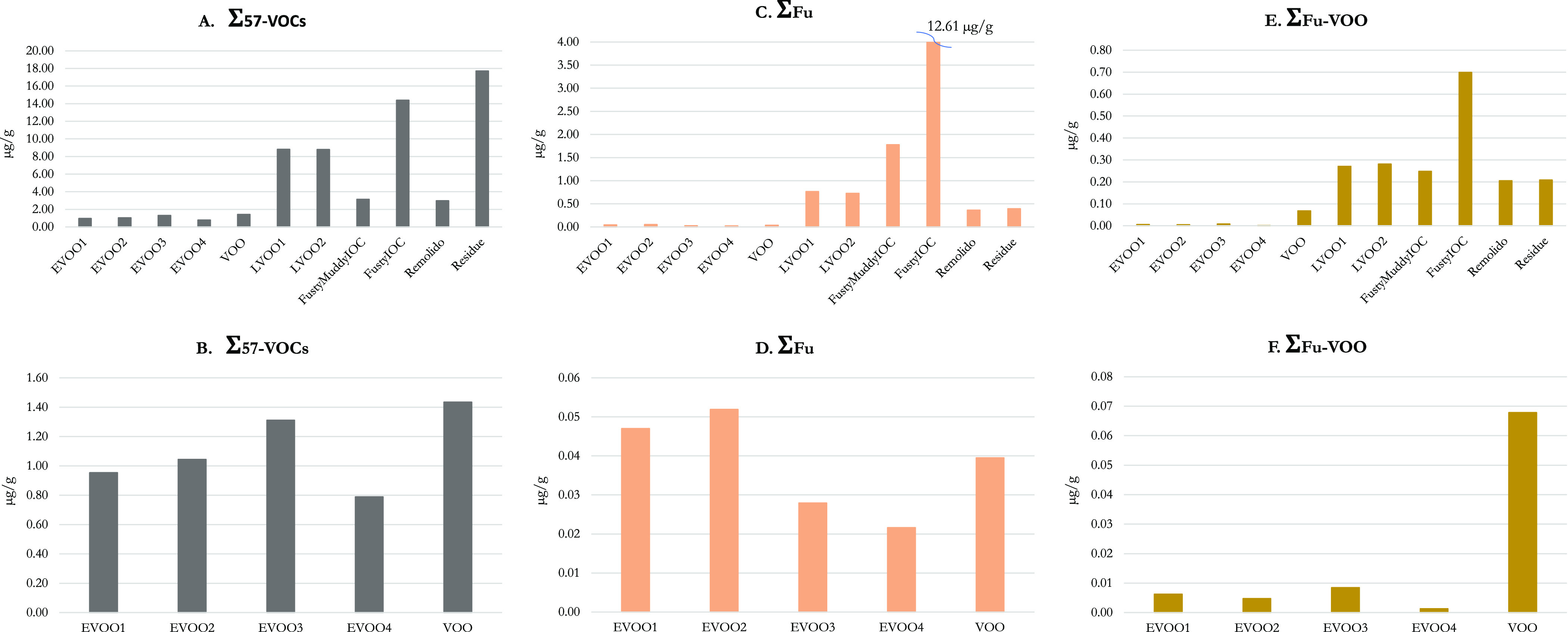
Sum of specific groups of VOCs in the analyzed olive oils. In particular, (A,B) Σ_57-VOCs_ is the sum of 57 VOCs associated with biological phenomena according to their evolution in open and closed alperujo over time; (C,D) Σ_Fu_ is the sum of VOCs more abundant in IOC reference than in other samples; (E,F) Σ_Fu-VOO_ is the sum of VOCs capable of distinguishing EVOO from VOO samples defective for fusty/muddy sediment. For each group, the contents in all samples and a zoom for EVOO and VOO samples are reported

After applying the above criteria, 57 VOCs were selected, almost completely represented by alcohols (21 VOCs), esters (19, of which 7 acetates), and carboxylic acids (12). The sum of the concentrations of such VOCs (Σ_57-VOCs_) in each analyzed olive oil sample was calculated. The obtained results are shown in the bar chart of Figure 3A for all the analyzed oils with a zoom in Figure 3B for only the analyzed EVOO and VOO samples. The defective samples with a high intensity of the defect (particularly the LVOO, the fusty IOC, and the Residue samples) were well-distinguished from the EVOO samples (Figure 3A) by this parameter, which was instead not at all able to distinguish the EVOO from the VOO samples (Figure 3B). The identification of a so wide group of VOCs indicates that many VOCs linked to biological detrimental phenomena are present in oils with the fusty/muddy sediment defect and that they are almost all alcohols, carboxylic acids, and esters. However, the marker given by the sum of these 57 VOCs appeared to be not definitely suitable to identify the presence of defects from biological origin in borderline EVOO/VOO samples. Furthermore, a parameter given by the sum of 57 molecules cannot be proposed for routine analysis in testing laboratories. Thus, we searched, among the above 57 VOCs, for a reduced number of molecules linked to the fusty/muddy sediment defect and possibly also capable of discriminating EVOO from VOO samples.

First, we selected those VOCs abundant in the fusty IOC reference oil samples, and the marker Σ_Fu_ was defined as the sum of VOCs more abundant in IOC fusty reference oils (Table 3). It can be proposed as a marker of defects from biological origin in those oils with a quite intense fusty/muddy sediment defect (e.g., the lampante olive oils). It was constituted by 1 ketone and a similar number of alcohols (4), acids (5), and esters (6, none of which acetates). As highlighted by the bar chart in Figure 3C, the marker was clearly able to differentiate the fusty IOC reference samples from all the other samples; it was also able to discriminate the highly defective (i.e., the lampante ones) from the EVOO samples, but it was not able to distinguish the EVOO from the VOO samples (Figure 3D). It must be always borne in mind that IOC reference oils are usually real samples used for proficiency interlaboratory tasting, and thus, they are not stable over time and not reproducible over the years and cannot be considered definitive reference standards for specific defects.^46^ Consequently, we defined a further marker for defects of biological origin in oils with low-intensity fusty/muddy sediment defects (the Σ_Fu-VOO_ in Table 3). The VOCs for this marker were those ones present in concentrations lower than 150 ng/g in all the analyzed olive oils, greater than 1 ng/g in the VOO, and lower than 1 ng/g in at least 3 out of the 4 EVOO samples; they were represented by a prevalence of esters (10, two of which acetates) followed by 4 alcohols, 1 ketone, and 1-hydroxyketone. Figure 3E,F shows that the Σ_Fu-VOO_ marker is clearly able to differentiate among EVOOs and all the other samples, including VOO samples (i.e., it was approx. 1 order of magnitude more concentrated in the VOO than in the EVOO samples).

**Table 3:** List of the VOCs Proposed as Markers for Defects from Biological Origin^a^

marker	VOCs
Σ_Fu_	1-propanol, 1-butanol, 2-pentanol, 2-heptanol, ethyl propanoate, ethyl 2-methyl propanoate, ethyl pentanoate, methyl hexanoate, ethyl hexanoate, ethyl heptanoate, 2-octanone, propanoic acid, 2-methyl propanoic acid, butanoic acid, pentanoic acid, hexanoic acid
Σ_Fu-VOO_	1-propanol, 1-heptanol, 2-heptanol, 1-octanol, 3-methylbutyl + 2-methylbutyl acetate, pentyl acetate, ethyl propanoate, ethyl 2-methyl propanoate, ethyl butanoate, methyl 3-methyl butanoate, ethyl 3-methyl butanoate, methyl hexanoate, ethyl hexanoate, ethyl benzoate, 2-octanone, acetoin

a (i) Σ_Fu_ for oils with intense fusty/muddy sediment defect, (ii) Σ_Fu-VOO_ for oils with low-intensity fusty/muddy sediment defect (i.e., the oils were difficult to classify by the panel test).

Surely, some processes other than those occurring in the alperujo samples can occur in one or more of the situations leading to the development of the fusty/muddy sediment defect, and we cannot exclude that some other volatile molecules might contribute to the fusty/muddy sediment defect. However, the approach followed in this research pointed out that a wide group of volatile molecules mainly belonging to alcohols, carboxylic acids, and esters and present in quite different concentrations in different olive oils are responsible for olive oil defects due to detrimental biological phenomena. In particular, some VOCs present in low concentration and including esters, alcohols, and also two ketones but no carboxylic acids seem to be responsible for the low-intensity fusty/muddy sediment defect in VOOs. They were able to also discriminate between VOO with fusty/muddy sediment as the prevalent defect (i.e., the borderline ones) and EVOO samples.

The Σ_Fu-VOO_ marker given by these 16 molecules has been shown to be a useful index for detecting the presence of defects of microbiological origin in defective samples, even when the defect is not intense, as in the case of virgin olive oils, usually difficult to be classified by the panel test. Looking at the values of Σ_Fu-VOO_ in the oils obtained with the analytical conditions applied in this study, a value of 0.04 μg/g could be proposed as a limit to discriminate EVOO samples from VOO samples defective for the fusty/muddy sediment defect. In the next step of the research, which is out of the aim of this manuscript, Σ_Fu-VOO_ has to be validated applying it to a higher number of samples, and the quantitative method for the 16 VOCs included in the index has to be validated to make it definitely reliable. The application of the defined index, together with the other proposed volatile molecular markers for winey/vinegary or rancidity defects, would allow detecting the presence of the main sensory defects in virgin olive oils by means of only analysis of VOCs, even in the borderline cases (difficult for the panel test).

## Supporting Information Available

Table S1 reports the list of the detected volatile organic compounds in alperujo and olive oil samples. Figure S1 shows the evolution of aldehydes in freeze-dried alperujo samples. Figure S2 shows the amounts of 1-hexanol, (*E*)-2-hexen-1-ol, and the related acetates in the olive oil samples (PDF)

## ABBREVIATIONS

EIC: extract ion chromatogram
EVOO: extra virgin olive oil
HS-SPME-GC–MS: head space-solid phase microextraction-gas chromatography–mass spectrometry
IOC: International Olive Council
ISTD: internal standard
LOX: lipoxygenase
LVOO: lampante virgin olive oil
VOCs: volatile organic compounds
VOO: virgin olive oil
