# Supplementary material for: Volatile Profile of Two-Phase Olive Pomace (Alperujo) by HS-SPME-GC–MS as a Key to Defining Volatile Markers of Sensory Defects Caused by Biological Phenomena in Virgin Olive Oil
Source: J Agric Food Chem. 2021 Apr 27;69(17):5155–66. doi: 10.1021/acs.jafc.1c01157 (PMC8278492; doi:10.1021/acs.jafc.1c01157)

Supporting information file of the manuscript:

Volatile Profile of Two-Phase Olive Pomace (Alperujo) by HS-SPME-GC-MS as a Key to Defining Volatile Markers of Sensory Defects Caused by Biological Phenomena in Virgin Olive Oil

Lorenzo Cecchi[1], Marzia Migliorini[2], Elisa Giambanelli[2], Anna Cane[2], Nadia Mulinacci[1*], Bruno Zanoni[3]

[1] Department of NEUROFARBA, University of Florence, Via Ugo Schiff 6, 50019 Sesto F.no, Florence, Italy

[2] Carapelli Firenze S.p.A., Via Leonardo da Vinci 31, Tavarnelle Val di Pesa, 50028, Firenze, Italy.

[3] Department of Agricultural, Food and Forestry Systems Management (DAGRI), University of Florence, Piazzale Delle Cascine 16, 50144, Florence, Italy.

*Corresponding author: Nadia Mulinacci

Dipartimento di NEUROFARBA, Università degli Studi di Firenze, Via Ugo Schiff 6, 50019 Sesto F.no (Firenze), Italy.

Tel.: +39 055 4573773 E-mail: nadia.mulinacci@unifi.it

Table S1. List of the detected volatile organic compounds in alperujo and olive oil samples; the symbol ***** indicates that the molecule was detected in trace amount. All the standards used for identification of the VOCs identified with commercial standard ("STD" in the column identification) are from Sigma Aldrich, Steinheim, Germany; the purity of the standard is given in the column "purity"

n°	Identification[a]	Compound	Purity (%)	RI_{CAL}[b]	RI_{REF}[c]	Nominal mw	quant/qual ions[d]
**1**	STD	Heptane	≥99.8	700	700	100	100/71
**2**	Tentative	Acetaldehyde	-	713	715	44	44/43
**3**	STD	Octane	≥99.7	800	800	114	*85/71*
**4**	Tentative	2-Methylpropanal	-	810	810	72	72/43
**5**	STD	Methyl acetate	≥99.9	829	839	74	*74/43*
**6**	STD	Ethyl acetate	≥99.9	902	898	88	*70/61*
**7**	STD	Methanol	≥99.9	911	910	32	31/29
**8**	STD	Butanone	≥99.9	914	915	72	*72/57*
**9**	STD	Methyl propanoate	≥99.9	918	908	88	88/59
**10**	STD	2-Methylbutanal	≥95	924	916	86	41/57-58
**11**	STD	3-Methylbutanal	≥97	929	912	86	44/71
**12**	STD	Ethanol	96	943	933	46	*46/31*
**13**	STD	Ethyl propanoate	≥99.7	969	957	102	*102/75*
**14**	Tentative	Ethyl 2-methylpropanoate	-	977	960	116	43/116
**15**	Tentative	2,3-Butanedione	-	988	970	86	43/86
**16**	STD	Pentanal	≥97.5	991	980	86	44/58
**17**	STD	3-Pentanone	100	992	983	86	57/86
**18**	Tentative	Methyl isobutyl ketone	-	1017	1012	100	*100/85*
**19**	Tentative	Isobutyl acetate	-	1023	1029	116	*56/73*
**20**	STD	2-Butanol *****	≥99.8	1025	1030	74	45/59
**21**	Tentative	Methyl 3-methylbutanoate	-	1028	1022	116	74/101
**22**	Tentative	2,2-Dimethyl-1-propyl acetate	-	1034	nd	130	43/75
**23**	STD	1-Penten-3-one	≥97	1034	1024	84	55/84
**24**	STD	1-Propanol	99	1044	1045	60	31/59
**25**	STD	Ethyl butanoate	≥99.5	1046	1041	116	88/60
**26**	Tentative	Toluene	-	1054	1058	92	*63/91*
**27**	Tentative	Ethyl 3-methylbutanoate	-	1074	1072	130	88/85
**28**	STD	Butyl acetate *****	≥99.9	1081	1075	116	43/56
**29**	STD	Hexanal	≥95	1093	1088	100	*72/44*
**30**	STD	2-Methyl-1-propanol	≥99.8	1096	1093	74	*33/74*
**31**	Tentative	2,2-Dimethyl-1-propanol	-	1112	nd	88	*73/57*

**32**	Tentative	3-Pentanol	-	1113	1124	88	59/31
**33**	Tentative	(*Z*)-2-Pentenyl acetate	-	1114	nd	128	*68/86*
**34**	STD	2-Pentanol	≥97	1124	1120	88	45/55
**35**	Tentative	2-Methylbutyl acetate + 3-methylbutyl acetate	-	1129	1128	130	43/70
**36**	Tentative	Ethyl pentanoate	-	1142	1139	130	88/101
**37**	STD	(*E*)-2-Pentenal	≥95	1144	1134	84	84/55
**38**	Tentative	1-Butanol	-	1148	1146	74	56/31
**39**	STD	1-Penten-3-ol	≥98	1163	1158	86	57/31
	**ISTD**	4-Methyl-2-pentanol 45	≥98	1167	1172	102	45/69
	**ISTD**	4-Methyl-2-pentanol 69	≥98	1167	1172	102	*69/45*
**40**	Tentative	Pentyl acetate	-	1180	1176	130	*70/43*
**41**	STD	2-Heptanone	≥99	1192	1185	114	58/71
**42**	Tentative	Methyl hexanoate	-	1195	1197	130	74/99
**43**	STD	Heptanal	≥97	1196	1197	114	*96/81*
**44**	STD	Limonene	≥99	1208	1210	136	93/136
**45**	STD	2-Methyl-1-butanol+3-methyl-1-butanol	≥98 - ≥98.5	1209	1210	88	70/41
**46**	STD	(*Z*)-3-Hexenal	50% in triacetin	1214	1204	98	*83/98*
**47**	STD	(*E*)-2 Hexenal	≥97	1233	1230	98	*83/98*
**48**	Tentative	Ethyl 3-methyl-2-butenoate	-	1236	1217	128	*100/128*
**49**	Tentative	2-Pentylfuran *	-	1239	1233	138	*138/82*
**50**	Tentative	Ethyl hexanoate	-	1240	1239	144	88/99
**51**	Tentative	Ethyl tiglate *	-	1246	1244	128	*113/128*
**52**	STD	1-Pentanol	≥99.8	1252	1254	88	*70/55*
**53**	Tentative	Styrene	-	1272	1267	104	104/103
**54**	STD	Hexyl acetate	≥99.7	1279	1269	144	43/84
**55**	STD	2-Octanone	≥99.5	1293	1287	128	58/128
**56**	Tentative	Methyl heptanoate	-	1294	1292	144	74/113
**57**	STD	Octanal	≥98	1299	1295	128	84/110
**58**	Tentative	3-Hydroxy 2-butanone (acetoin)	-	1301	1298	88	*88/45*
**59**	Tentative	Ethyl (*Z*)-3-hexenoate	-	1308	1295	142	69/142
**60**	STD	1-Octen-3-one	≥97	1313	1305	126	55/83
**61**	STD	(*E*)-2-Penten-1-ol	95	1314	1316	86	57/86
**62**	Tentative	1-Hydroxy-2-propanone	-	1317	1318	74	*74/43*
**63**	STD	2-Heptanol	≥98	1318	1318	116	45/55
**64**	STD	(*Z*)-2-Penten-1-ol	95	1322	1321	86	57/68
**65**	STD	(*Z*)-3-Hexenyl acetate	≥98	1325	1320	142	*82/67*
**66**	STD	(*E*)-2-Heptenal	≥95	1338	1334	112	*83/70*

**67**	STD	(*E*)-2-Hexenyl acetate *	98	1339	1337	142	*100/82*
**68**	Tentative	Ethyl heptanoate	-	1339	1334	158	88/113
**69**	STD	6-Methyl-5-hepten-2-one	≥97	1351	1347	126	*108/69*
**70**	STD	1-Hexanol	≥99.9	1354	1356	102	56/69
**71**	Tentative	2-Methyl-2,3-pentanediol	-	1359	nd	118	59/71
**72**	STD	(*E*)-3-Hexen-1-ol	97	1365	1364	100	*67/82*
**73**	Tentative	2- Hydroxy-3-pentanone	-	1373	1361	102	45/59
**74**	Tentative	Heptyl acetate	-	1380	1377	158	*70/98*
**75**	STD	(*Z*)-3-Hexen-1-ol	≥98	1388	1384	100	67/82
**76**	Tentative	Acetoin acetate	-	1389	1389	130	*87/43*
**77**	Tentative	Methyl octanoate	-	1397	1399	158	74/158
**78**	STD	2-Nonanone *	95	1398	1398	142	58/142
**79**	STD	Nonanal	≥99.5	1404	1401	142	57/98
**80**	STD	(*E*)-2-Hexen-1-ol	96	1408	1406	100	57/82
**81**	Tentative	(*E*)-4-Hexen-1-ol *	-	1413	1408	100	67/82
**82**	STD	2-Octanol *	≥97	1417	1412	130	*97/55*
**83**	STD	(*Z*)-2-Hexen-1-ol	95	1418	1410	100	57/82
**84**	STD	(*E,E*)-2,4-Hexadienal	≥95	1420	1411	96	81/96
**85**	Tentative	Ethyl octanoate	-	1443	1449	172	88/127
**86**	STD	(*E*)-2-Octenal	≥97	1447	1451	126	70/83
**87**	STD	1-Octen-3-ol	≥98	1451	1451	128	57/72
**88**	STD	1-Heptanol	≥99.9	1456	1458	116	70/56
**89**	STD	Acetic acid	≥99	1464	1479	60	43/60
**90**	Tentative	Methyl nonanoate	-	1499	1503	172	74/141
**91**	Tentative	4-Hepten-1-ol	-	1505	1502	114	81/114
**92**	STD	Decanal *	≥95	1510	1515	156	*112/43*
**93**	STD	(*E,E*)-2,4-Heptadienal	≥88	1512	1508	110	*110/81*
**94**	Tentative	Ethyl nonanoate	-	1536	1530	186	88/141
**95**	STD	Benzaldehyde	≥95	1544	1534	106	105/106
**96**	STD	(*E*)-2-Nonenal *	≥95	1547	1548	140	*96/83*
**97**	STD	1-Octanol	≥99.7	1558	1561	130	*31/56*
**98**	Tentative	Nonyl acetate	-	1582	1573	186	43/98
**99**	STD	Propanoic acid	≥99.8	1585	1564	74	74/73
**100**	Tentative	2-Methyl propanoic acid		1589	1571	88	43/88
**101**	Tentative	2,2-Dimethyl propanoic acid	-	1595	1586	102	57/41
**102**	Tentative	4-Hexen-2-one	-	1600	nd	98	43/98
**103**	Tentative	Methyl decanoate	-	1601	1593	186	74/143

**104**	Tentative	Ethyl decanoate	-	1644	1648	200	88/155
**105**	STD	Butanoic acid	≥99.5	1649	1637	88	60/73
**106**	Tentative	2,6-Dimethylhept-5-en-1-ol *****	-	1654	1654	142	69/109
**107**	STD	1-Nonanol	98	1659	1663	144	*31/56*
**108**	STD	(*E*)-2-decenal	≥96	1660	1650	154	*110/70*
**109**	Tentative	3-Methyl butanoic acid	-	1686	1686	102	60/87
**110**	Tentative	2-Methyl butanoic acid	-	1687	1681	102	74/57
**111**	Tentative	Ethyl benzoate	-	1688	1673	150	105/150
**112**	STD	(*E*,*E*)-2,4-Nonadienal	≥90	1718	1709	138	81/138
**113**	STD	Pentanoic acid	≥99.8	1754	1755	102	60/73
**114**	Tentative	1-Decanol	-	1758	1748	158	70/112
**115**	Tentative	(*E*)-2-Pentenoic acid	-	1815	nd	100	55/100
**116**	STD	(*E*,*E*)-2,4-Decadienal	≥84	1822	1815	152	81/152
**117**	STD	Hexanoic acid	≥99	1854	1852	116	60/73
**118**	STD	Guaiacol *****	99	1874	1861	124	109/124
**119**	Tentative	Benzyl alcohol	-	1887	1869	108	79/108
**120**	STD	2-Phenylethanol	99	1925	1924	122	91/122
**121**	Tentative	Heptanoic acid	-	1961	1950	130	60/73
**122**	Tentative	(*E*)-3-Hexenoic acid	-	1986	1957	114	55/114
**123**	STD	Phenol *****	99	2012	2028	94	94/66
**124**	STD	4-Ethylguaiacol *****	≥98	2047	2036	152	137/152
**125**	Tentative	Octanoic acid	-	2066	2052	144	60/73
**126**	Tentative	Nonanoic acid	-	2169	2162	158	60/73
**127**	STD	4-Ethyl-phenol	99.8	2179	2183	122	107/122

[a] Identification: STD, identification was confirmed with mass spectrum and retention index in accordance with the commercial standard; tentative, molecule was tentatively identified matching the mass spectrum with NIST08/Wiley98 library and the retention index with NIST Chemistry WebBook

[b] RI_{CAL}: non-isothermal Kovats retention indices from temperature-programming, using the definition of Van den Dool and Kratz, 1963.[38]

[c] RI_{REF}: non-isothermal Kovats retention indices from temperature-programming from Chemistry WebBook.

[d] italic indicates that the quantification of the molecule was based on an ion with intensity lower than 50% of the base peak

Figure S1. Evolution of aldehydes in freeze-dried alperujo samples. Data are the mean of three independent determinations and are expressed on dry matter basis, and the standard error of mean is also reported in the charts.

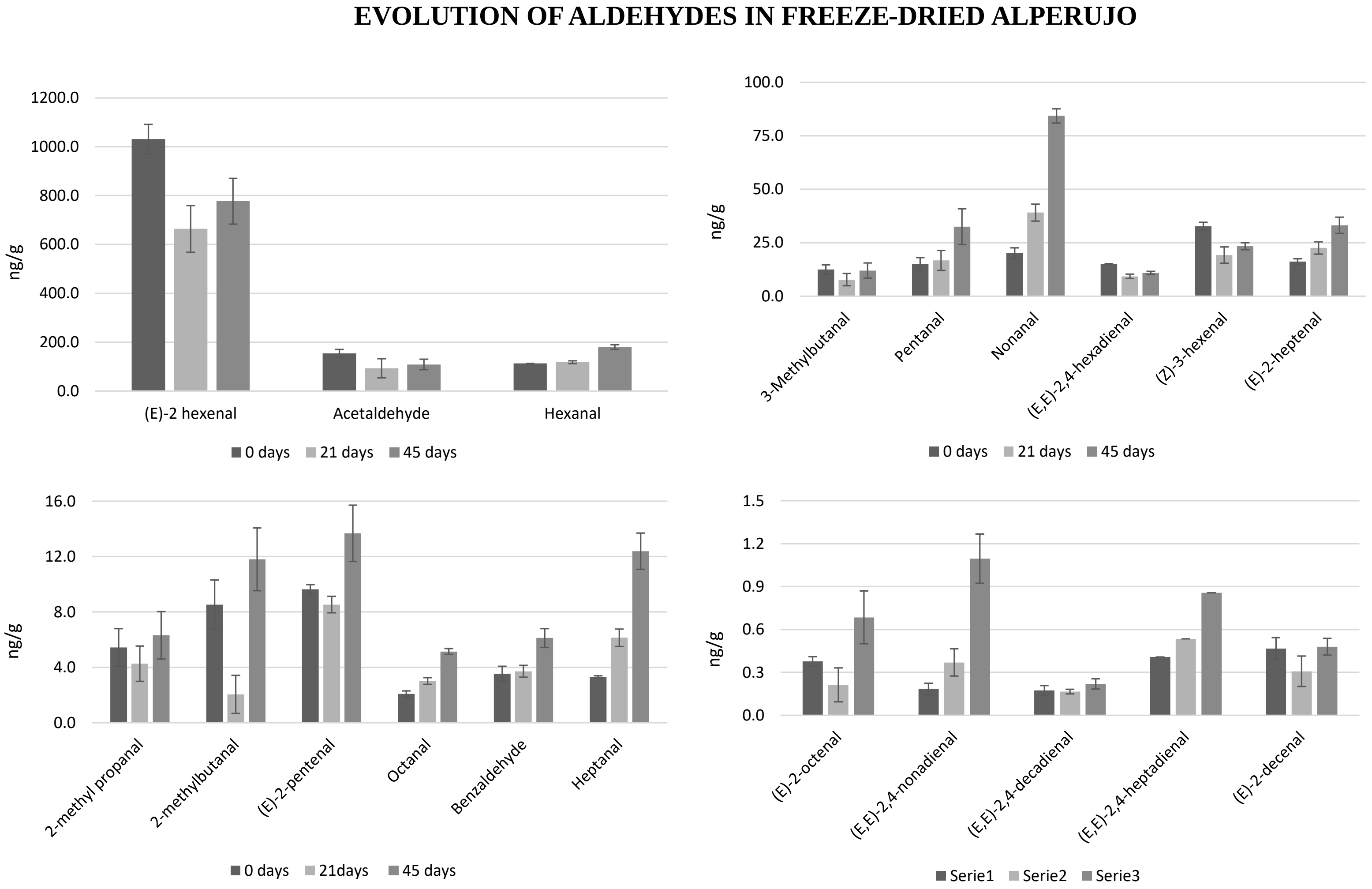

Figure S2. Amount of 1-hexanol, (*E*)-2-hexen-1-ol and the related acetates in the olive oil samples.

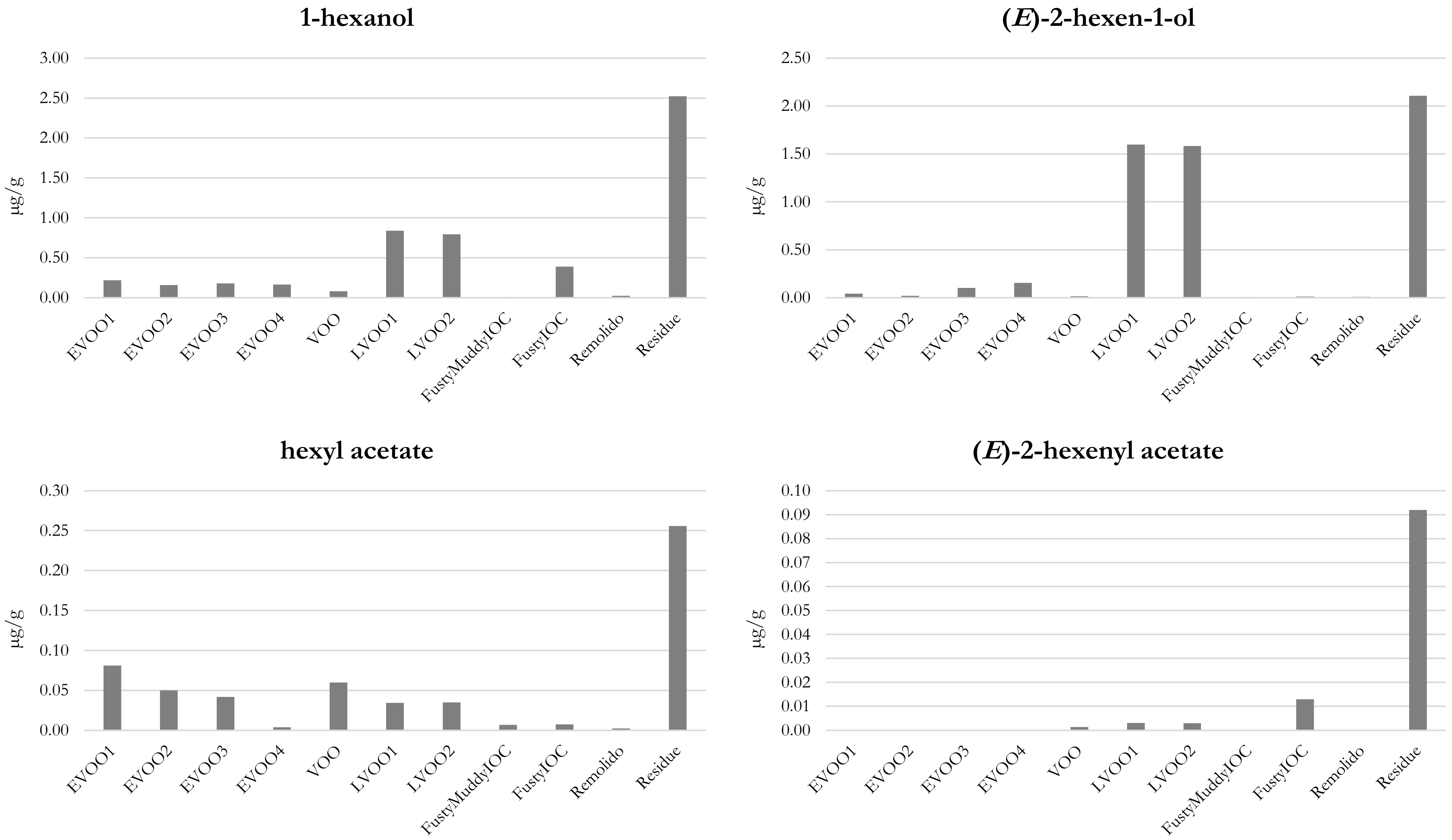

Supplement: Supplementary file 1 — jf1c01157_si_001.pdf [file jf1c01157_si_001.pdf]
